# Impact of Parvovirus B19 Viremia in Liver Transplanted Children on Anemia: A Retrospective Study

**DOI:** 10.3390/v9060149

**Published:** 2017-06-13

**Authors:** Michael Würdinger, Susanne Modrow, Annelie Plentz

**Affiliations:** 1 Institute of Medical Microbiology and Hygiene, University of Regensburg, 93053 Regensburg, Germany; michael.wuerdinger@usz.ch (M.W.); susanne.modrow@ukr.de (S.M.); 2 Department of Surgery and Transplantation, University Hospital of Zurich, 8091 Zürich, Switzerland

**Keywords:** parvovirus B19, children, pediatric, transplantation, anemia

## Abstract

Acute parvovirus B19 (B19V) infection in immunocompromised patients may lead to severe anemia. However, in adult transplant recipients, B19V reactivations without anemia and low-level viremia are common. The impact of B19V in pediatric transplant patients, with high risk of primary infection, is investigated here. In a six-month period, 159 blood samples of 54 pediatric liver transplant recipients were tested for B19V DNA by quantitative real-time PCR. Viremia was correlated with anemia and immunosuppression and compared with rates in adult transplant recipients. B19V DNA was detected in 5/54 patients. Primary B19V infections were observed in four patients prior to and in one patient after transplantation. Rates of viremia were significantly higher in pediatric recipients than in adults. Prolonged virus shedding after primary infection prior to transplantation accounts for most viremic cases. Anemia was significantly more frequent in samples from viremic patients, but remained mild. In 15% of anemic samples, B19V DNA was detected. Therefore, in anemic pediatric transplant recipients, diagnostics for B19V seem reasonable.

## 1. Introduction

In immunocompetent individuals, acute parvovirus B19 (B19V) infection is a mild disease associated with transient anemia, rash and arthritis. In contrast, in immunocompromised patients, cases of persisting B19 viremia with severe life-threatening chronic anemia have been reported [1,2,3]. Those clinical courses occur in acute infections with high B19V DNA levels after solid organ transplantation, whereas B19V reactivations are more likely in adult transplant recipients and lead to low-level DNAemia, which is not associated with notable anemia [4,5]. Therefore, B19V does not seem to be a frequently harmful pathogen in adult transplant recipients. However, children are at higher risk of a primary B19V infection due to the low level of immunoglobulin G (IgG) seroprevalence [6]. It is unclear if there is a higher rate of acute B19V infections and consequently severe anemia in pediatric transplant recipients since larger studies are lacking.

The aims of this retrospective anonymous study were: (1) to assess the prevalence and quantity of B19V DNA, to determine the respective B19V genotypes and the serostatus at liver transplantation among pediatric patients, and (2) to correlate the occurrence of B19 viremia with hemoglobin levels and reticulocyte counts as surrogate markers of pure red cell aplasia and to compare these findings with previously published data on B19V prevalence in adult transplant recipients [4].

## 2. Materials and Methods

For retrospective analysis, pediatric patients between 0 and 18 years who had undergone liver transplantation in the pediatric university hospital of Regensburg between 2008 and 2012 were eligible for the study. Remaining samples from routine blood analyses derived from a period of six months after transplantation were tested for B19V DNA. Follow-up samples were selected at intervals of 30 days or as close as possible to these dates. In case of re-transplantation follow-up was extended or extra cases were defined. All samples were analyzed for B19V DNA (genotypes 1–3) by quantitative real-time PCR. Hemoglobin levels and reticulocyte counts of each analyzed sample were recorded. For assessment of the patients’ serostatus, B19V IgG was tested in pre-transplantation samples or, if not available, shortly after transplantation. Because of intravenous immunoglobulins (IVIG) routinely transfused in a large number of patients for cytomegalo virus (CMV) prophylaxis, which contributes to the serostatus for several months, B19V IgG negative patients were tested again at one month to assess the postoperative serostatus. Clinical data on underlying disease, immunosuppression, IVIG transfusions and symptoms characteristic of B19V infection were obtained from the patient records. All data were pseudonymized. The study was approved by the Ethics Committee of the University Hospital of Regensburg on 31 March 2011 (reference number 11-101-0036).

B19V DNA was amplified using an in-house real-time quantitative TaqMan PCR assay including all three genotypes according to the previously published protocol [4]. Purified B19V plasmids were included as positive controls and for calibration and B19V-seronegative samples as negative controls. The detection limit of the assay, defined as the DNA concentration with at least 95% of positive samples detectable, was 600 genome equivalents (geq)/mL (215 IU/mL) for each genotype. Reproducible positive results below the detection limit (<600 geq/mL) were described as ‘weakly positive’.

B19V specific IgG, immunoglobulin M (IgM), and IgG avidity were analyzed by enzyme immune assay (EIA) and immunoblot according to the manufacturer’s instructions (recomWell parvovirus B19 IgG and IgM assay, Mikrogen, Neuried, Germany).

Reference levels of the Department of Clinical Chemistry and Laboratory Medicine of the University Hospital of Regensburg were used for definition of anemia (Table 1).

## 3. Results

### 3.1. Patients

A total of 51 pediatric liver transplant recipients met the inclusion criteria. Due to re-transplantations, a total of 54 cases were analyzed. The median age was two years, 56% were female, 44% male. Thirty-six liver transplantations were performed in infants, 15 patients where six years or older. In patients in infancy almost 2/3 suffered from biliary atresia, whereas patients aged six years or older mostly suffered from cystic fibrosis and autoimmune hepatitis. A total of 159 samples were available for analysis.

### 3.2. B19V DNA Detection

Within the first six months post-transplantation (post-Tx), in 12 samples (7.6%) from five patients (9.3%) B19V DNA was detected after transplantation, in all cases B19V genotype 1. In 7/12 samples, the B19V DNA load was <600 geq/mL, >10^3^ geq/mL B19V-DNA/genotype 1 were detectable in four and >10^5^ geq/mL in one sample. In 3/5 (60%) of these patients, initial B19V DNA detection post-Tx was at month 1, in one patient at month 3. In these four patients B19V DNA was already found in pre-transplantation (pre-Tx) specimens, as well. In one pre-Tx DNA-negative patient initial B19V DNA detection was at month 5, three days after re-transplantation (month 0). Thus, in all cases viremia was observed within the first three months after transplantation.

### 3.3. B19V IgG Serostatus

B19V IgG was detected in 31.5% (17/54 patients) pre-Tx. In patients younger than one year, 5/20 were seropositive (0–4 months old). The four patients with viremia pre-Tx were also B19V IgG positive. That means that 4 (23.5%) of 17 children with an earlier infection were still viremic. As five patients had probably maternal antibodies, this number is raised to 4/12 (33.3%). In month 1, 70.4% (38/54) were seropositive due to IVIG.

### 3.4. Dependence of Viremia on Immunosuppressive Regimen

In all cases an induction therapy with basiliximab was implemented. Mostly, immunosuppressive regimens were used combining prednisolone with cyclosporine (56%) or tacrolimus (20.8%). Changes of medication from cyclosporine to other immunosuppressive drugs were performed in the further course of the observation period. Thus, we compared the immunosuppressive regimens with B19V viremia in each sample. B19V viremia was more frequent in tacrolimus-based than in cyclosporine-based regimens (*p* = 0.0003): B19V viremia was observed in 10/50 tacrolimus-based regimens (20%), whereas viral genomes were found only in 2/106 cyclosporine-based regimens (1.9%).

Furthermore, the correlation of B19V viremia with high dose prednisolone therapy (≥60 mg/m^2^/day) used for treatment of acute rejection (13 patients, one patient twice) or for initiation of immunosuppression (two patients) was evaluated. In 3/15 samples, B19V viremia was detectable simultaneously to high-dose prednisolone administration (20%), whereas B19V DNA could be found only in 9/144 of the remaining samples (6.3%). This difference was significant in Fisher’s exact test (*p* = 0.036).

### 3.5. Association of Viremia and Hemoglobin Levels

Anemia was found in 17/27 samples (63.0%) from patients with B19V viremia, in contrast only in 43/132 samples (32.6%) of all 49 B19V negative patients. Also, anemia was more frequent in all B19V DNA positive (9/12, 75%) samples than in all B19V DNA negative samples (50/145, 34.5%). Both comparisons were highly significant (*p* = 0.003 and 0.006). There were also higher rates of reticulocytopenia in viremic patients (20.0% vs. 14.3%) and samples (33.3% vs. 13.4%), respectively. Due to low number of samples with reticulocyte counts, the significance of these findings remains unclear.

### 3.6. Case Report of a Primary B19V Infection after Pediatric Liver Transplantation

Patient PLTX_19, a three-year old boy suffering from progressive familial intrahepatic cholestasis, primary B19V infection occurred after liver transplantation. He underwent re-transplantation after 143 days due to acute refractory rejection. B19V IgG was lacking despite IVIG transfusion after first transplantation. He received high doses of prednisolone (≥60 mg/m^2^/day) and a tacrolimus-based maintenance therapy in temporal relation to re-transplantation.

**Figure 1:**
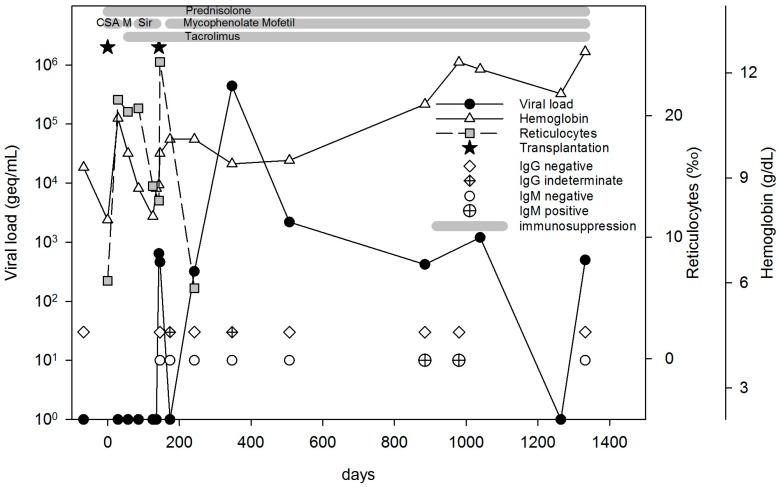
Course of patient PLTX_19. For more than three years after initial detection, B19V DNA was detectable with a maximal value of 10^5^ geq/mL at month 7 after re-transplantation. During this period, the patient was B19V IgG negative or indeterminate and became transiently B19V IgM positive two years after re-transplantation. Anemia was present only two months before initial detection of B19V DNA with hemoglobin levels between 7.9 g/dL and 8.7 g/dL. Reticulocytopenia (6‰) was detected only once in month 4 after second transplantation with low viral load at the same time. CSA—Cyclosporine A. M—Mycophenolat Mofetil. Sir—Sirolimus. IgM—immunoglobulin M.

Parvovirus B19 DNA (<600 geq/mL) was detected for the first time three days after re-transplantation. Unfortunately, neither the donor organ nor donor blood was available for testing, so it remains unknown if the organ was the source of infection. The patient developed an itching exanthema with target lesions and slapped cheeks two days after transplantation, consistent with the clinical manifestation of the fifth disease. The course of viremia and other markers are depicted in Figure 1.

### 3.7. Comparison of Pediatric and Adult Transplant Recipients Regarding Clinical Impact of B19V DNA Detection

The number of B19V DNA positive samples was significantly higher in pediatric than in adult liver transplant patients (10.3% vs. 2.7%, *p* < 0.001) [4]. Also, the prevalence of B19 viremia was higher in pediatric than adult liver transplant recipients (9.3% vs. 5.5%, not significant.).

In pediatric liver transplant patients, the overall rate of anemia was significantly lower than in adult liver transplant recipients (37.7% vs. 81.5%, *p* < 0.001). However, in pediatric patients, 15.3% (9/59) of anemic samples simultaneously showed B19Viremia, whereas in adult liver transplant patients this constellation was observed only in 2.6% (7/269) (*p* < 0.001).

## 4. Discussion

In this first larger study on B19V in pediatric transplant patients, a B19V DNA prevalence of 9.3% was found, which is markedly higher than previously estimated on the basis of case reports [7]. There were higher B19V DNA loads in pediatric patients with most likely a recent primary infection pre-Tx [8,9,10] than in adult patients with reactivation [4]. In all patients, the B19V genotype 1 was found, the most widely-spread genotype [11].Prolonged viremia after primary infection prior to transplantation had a wider impact on anemia than reactivations after transplantation [4]. The level of anemia seems to correlate with B19V DNA loads, since there is no anemia found in adult patients with low-level B19V DNAemia [4,5], mild anemia in our pediatric collective with moderate viremia, and severe anemia in immunocompromised patients with high-level viremia [1,2,12,13,14,15]. The overall rate of anemia in pediatric liver transplant recipients was distinctly lower than in adult transplant patients (37.7% vs. 84.2%) [4]. Lower rates of anemia in pediatric liver transplant patients are known [16], the reasons remain unclear. It is remarkable that as many as 15.3% of samples with anemic hemoglobin levels were simultaneously B19V DNA positive, similar to 19.2% in anemic pediatric liver transplant recipients in a smaller collective [17]. Thus, B19V viremia can be regarded a major risk factor for anemia in pediatric liver transplant recipients, in contrast to adults.

As many as one-third of all children with a former infection were still viremic at the time point of transplantation and thus at risk for anemia. IVIG was routinely transfused in CMV high-risk patients and in under-six-month-olds, which contributes to transient immunity [18,19,20]. This raised the seroprevalence from 31.5% pre- to 70.4% post-Tx which is similar to that of adult transplant recipients [4] and led to a low incidence of primary infections after transplantation (1/54, 1.9%), comparable to that in adult transplant recipients (0.7%) [4].

The rate of B19V viremia in patients treated with tacrolimus-based regimens was significantly higher than in patients treated with cyclosporine-based regimens. Other case reports also show pure red-cell aplasia due to B19V infection after application of tacrolimus—in one case the infection remitted only after switching to cyclosporine [21,22,23] as the immunosuppressive effect is lower [24,25]. Also BK virus infections occur more frequently in patients with tacrolimus-based immunosuppression [26]. High doses of prednisone also led to a significantly higher rate of B19V infections, consistent with a straight increase of CMV infection risk with cumulative methylprednisolone-dose [27].

There was one unusual case of a primary B19V infection post-Tx with an unknown source of infection. Surprisingly, he presented with atypically low DNA-loads (<600–440,000 geq/mL), negative antibodies, and did not have anemia at all, but showed an exanthema consistent with fifth disease two days after transplantation. This manifestation is the result of immune complex formation [28], but in this case, viral load was much too low to build enough immune complexes, specific IgG and IgM were not detectable. As well, it remains unclear why high viral loads and prolonged pure red-cell aplasia did not occur, as described before in several case reports [2,29,30]. This case and another one in a heart transplanted adult [4] obviously show that not every primary infection necessarily leads to a severe course of infection.

In this study, parvovirus B19V viremia most often occurred as a prolonged shedding of virus after a primary infection prior to transplantation. It caused mild anemia. A primary infection post-Tx surprisingly did not result in a severe course. However, as 15% of anemic samples were B19Viremic, we recommend performing diagnostics for B19V DNA in anemic pediatric transplant recipients though routine screening after transplantation does not seem to be necessary.

## Figures and Tables

**Table 1:** Characteristics of parvovirus B19 (B19V) DNA positive patients.

Patient ID, Gender, Age (Years), Donor	Day Post-Tx	DNA (geq/mL)	gt	IgG	IgG-Quantity ^1^	Hb (g/dL)	Reti (‰)
PLTX_02, m, 13, D	−13	9200		pos	+++		
	34	<600	1	pos	+++	10.3 *	nd
	70	<600				10.5 *	nd
	98	<600				10.6 *	nd
	164	0		pos	+++	9.4 *	nd
PLTX_03, f, 11, D	−148	1700		pos	+++		
	30	680	1	pos	+++	7.9 *	nd
	59	<600		pos	+++	10.4 *	nd
	90	0				10.3 *	nd
	128	0				10.3 *	nd
	158	0				9.3 *	nd
	168	0				10.9	nd
PLTX_08, m, 10, D	0	1300		pos	+++		
	32	0				8.3 *	33
	59	0				nd	nd
	92	<600	1	pos	+++	10.1 *	nd
	118	0		pos	+++	nd	nd
	146	0				12.0	nd
	172	0				11.9	nd
PLTX_19, m, 3, D	−67	0		neg	−−		
(Re-Tx: day 143, D)	29	0				10.7	21
	57	0				9.7	20
	86	0				8.7 *	21
	126	0				7.9 *	nd
	146	<600	1	neg	−−	9,7	24
	174	0		i	−/+	10.1	nd
	242	<600		neg	−−	10.1	6 *
	347	440,000		i	−/+	9.4	nd
PLTX_28, f, 10, D	1	5100		pos	+++	9.5 *	14
	30	3200	1	pos	+++	10.1 *	23
	58	3600				9.3 *	24
	129	3000		pos	++	8.9 *	3 *

pos—positive; neg—negative; i—indeterminate; nd—not detected; m—male; f—female; D—deceased donor; Hb—hemoglobin levels; Reti—reticulocyte counts. * levels indicating anemia or reticulocytopenia (age dependent standard hemoglobin levels [g/dL]: 0–1 day: 17.7–26.5; 2–7 days: 16.2–25.5; 8–30 days: 10.1–24; 31–59 days: 9.2–18; 60–365 days: 9–14.6; 366–730 days: 9.1-15; 2–9 years: 9.2–15.5; 10–11 years: 10.7–16.5; 12–13 years: 10.8–16.2; 14–17 years: 11–15.9; 18–20 years (f): 11.2–15.7, 18–20 years (m): 13.7–17.5). ^1^ semiquantitative presentation: indeterminate: −/+; 24.1–49.9 U/mL: +; 50.0–199.9 U/mL: ++; >200 U/mL: +++. Re-Tx: second transplantation; post-Tx: post transplantation; gt: genotype; IgG: immunoglobulin G.
